# Effect of increasing heart rate on finger photoplethysmography fitness index (PPGF) in subjects with implanted cardiac pacemakers

**DOI:** 10.1371/journal.pone.0207301

**Published:** 2018-11-28

**Authors:** Amilia Aminuddin, Isabella Tan, Mark Butlin, Alberto P. Avolio, Hosen Kiat, Edward Barin, Nor Anita Megat Mohd Nordin, Kalaivani Chellappan

**Affiliations:** 1 Department of Physiology, Universiti Kebangsaan Malaysia Medical Center, Kuala Lumpur, Malaysia; 2 Faculty of Medicine and Health Sciences, Macquarie University, Sydney, Australia; 3 Faculty of Medicine, University of New South Wales, Sydney, Australia; 4 Centre of Advance Electronic & Communication Engineering (PAKET), Universiti Kebangsaan Malaysia, Bangi, Selangor, Malaysia; Indiana University, UNITED STATES

## Abstract

Finger photoplethysmography (PPG) is a noninvasive method that measures blood volume changes in the finger. The PPG fitness index (PPGF) has been proposed as an index of vascular risk and vascular aging. The objectives of the study were to determine the effects of heart rate (HR) on the PPGF and to determine whether PPGF is influenced by blood pressure (BP) changes. Twenty subjects (78±8 years, 3 female) with permanent cardiac pacemakers or cardioverter defibrillators were prospectively recruited. HR was changed by pacing, in a random order from 60 to 100 bpm and in 10 bpm increments. At each paced HR, the PPGF was derived from a finger photoplethysmogram. Cardiac output (CO), stroke volume (SV) and total peripheral resistance (TPR) were derived from the finger arterial pressure waveform. Brachial blood pressure (BP) was measured by the oscillometric method. This study found that as HR was increased from 60 to 100 bpm, brachial diastolic BP, brachial mean BP and CO were significantly increased (p<0.01), whilst the PPGF and SV were significantly decreased (p<0.001). The effects of HR on the PPGF were influenced by BP, with a decreasing HR effect on the PPGF that resulted from a higher BP. In conclusion, HR was a significant confounder for PPGF and it must be taken into account in analyses of PPGF, when there are large changes or differences in the HR. The magnitude of this effect was BP dependent.

## Introduction

The assessment of a peripheral pulse as a marker for vascular ageing and vascular risk has gained increasing popularity as the technique is simple, noninvasive, reliable and accurate. One method that is used to assess a peripheral pulse is the finger photoplethysmography (PPG). A signal from a PPG can readily be obtained by placing a pulse oximeter probe on a finger, which has a high degree of superficial vasculature. Signals from a PPG represent relative blood volume changes in the peripheral circulation of each cardiac cycle. The PPG pulse contour consists of a rising edge, a primary peak, a secondary peak and a dicrotic notch. The shape of the pulse is largely influenced by the interaction of several factors, such as a ventricular ejection, arterial stiffness, arterial wave reflection and arterial resistance. Furthermore, other physiological factors, such as sympathetic activity and environmental factors, such as temperature, may have an additional impact on the PPG wave contour [1].

By analyzing the shape of the pulse, previous studies have derived several parameters that are related to vascular aging, such as pulse transit time (PTT), stiffness index, reflection index, as well as the second derivative properties of the PPG [2, 3, 4]. Based upon analyses of the PPG pulse contour, Chellappan et al. introduced an index that can measure vascular fitness by using a PPG, termed as PPG fitness index (PPGF), which has been proposed as an index of vascular risk [5]. This index was further explored in the Malaysian population for quantification of vascular ageing [6]. The PPGF was derived based upon a morphological investigation by comparing age and the disease classified PPG pulse with a reference pulse (the PPG pulse of a clinically verified healthy 19-year-old subject). A previous study has found that the PPGF was inversely related with age and it was lower in those with cardiovascular risk factors when compared to healthy subjects at a similar age [6].

Heart rate (HR) is an important physiological factor that can affect vascular properties, such as aortic compliance and arterial wave reflection [7, 8, 9]. An increased HR is related to shorter duration of systolic and diastolic times, the late arrival of wave reflection in the aorta from the periphery [9] in relation to ejection duration and increased arterial stiffness [8, 10, 11]. However, the impact of HR changes on the PPGF has yet to be investigated. This current study has hypothesized that there is an association between HR and the PPGF, since HR influences other characteristics of a vascular function, as previously mentioned. These changes may be dependent on changes in blood pressure (BP) or other related factors.

## Methodology

This study obtained ethical approval from the Human Ethics Committee of Macquarie University, Sydney, NSW, Australia and a signed consent was obtained from all of the participants. Subjects with implanted pacemakers or implantable cardioverter defibrillators were recruited from the Cardiac Health Institute and the Macquarie Heart clinics at Macquarie University. The inclusion criteria involved a spontaneous rhythm below 60 bpm (bradycardia) due to several types of diseases, such as sick sinus syndrome, heart block and dilated cardiomyopathy. The exclusion criteria involved uncontrolled heart failure, recent myocardial infarction and unstable angina. It was calculated that in order to detect a 2% change in the PPGF, arising from the result of a standard deviation of 5.66% at an alpha level of 5% and with a power of 80%, a minimum sample of 15 subjects would be required [12]. A total of twenty consecutive patients that fulfilled the entry criteria were included in the study.

### Measurements of height and weight

Height was measured barefoot via a measurement tape attached to the wall. Weight was measured via a weighing scale (SECA, Hamburg, Germany). Body mass index was calculated as weight/height^2^ (kg/m^2^).

### Measurements of Brachial blood pressure, stroke volume, Cardiac output and total peripheral resistance

A brachial cuff was placed on the right arm and brachial BP was measured once by the oscillometric method (SphygmoCor XCEL, AtCor Medical, Sydney, Australia) whilst in a supine position. Measurements of stroke volume (SV) and total peripheral resistance (TPR) were obtained from finger arterial pressure waveform by using the Model flow method (Finometer PRO, Finapres Medical Systems, Amsterdam, The Netherlands) [13]. Cardiac output (CO) was calculated as a product of HR and SV. Electrocardiogram (ECG) was also acquired continuously for the duration of the study for monitoring of HR (PowerLab acquisition system, LabChart software, ADInstruments, Dunedin, New Zealand)

### PPGF recording

All measurements were performed in a room with a controlled temperature of between 20–25° C. The subjects were asked to abstain from coffee, fatty meals and smoking, for at least 4 hours prior to the study. The investigation was conducted with the subjects in a supine position. A PPG probe (Nellcor^TM^ probe) was attached to the right index finger of the subject and the resulting signals were henceforth obtained. The system was connected to a desktop computer that was running the processing software (PulseBox, Rapid Labs Sdn. Bhd, Malaysia). This software has been validated and the repeatability of the PPGF has been previously studied [14]. The sampling rate was 275 Hz and the resolution was 16 bits [5]. The data processing was performed off-line. The PPGF was derived from an AC component of the PPG morphological changes and compared against a healthy 19-year-old reference (gender specific) who was identified from the population. It was calculated by using this formula [14]:

$$PPGF=100\times \left(1-\frac{\sqrt{\sum \left((x-\bar{x}\right)-(y-\bar{y}){)}^{2}}}{\sqrt{\sum (y-\bar{y}{)}^{2}}}\right)$$

where x was the reference pulse amplitude, and y was the tested pulse amplitude.

Vascular age was estimated based upon age versus the PPGF from the regression model that was established by using the Malaysian population data [6].

### Pacing protocol

The PPGF was acquired over 60 seconds followed by measurements of SV and TPR. Brachial BP was measured by a SphygmoCor XCEL device, as previously mentioned. All measurements were repeated at five different paced HR levels (60, 70, 80, 90, and 100 bpm) that were set in a random order, in order to avoid a time-based bias. These measurements were recorded after one minute of pacing, so as to allow for stable hemodynamic data. The pacing protocol has been previously described and validated [9].

### Statistical analyses

Normality was assessed by the Shapiro-Wilk test. All of the data was normally distributed. The data is presented as mean ±SD, unless otherwise stated. The data for the PPGF, brachial BP, SV, CO and TPR at each heart rate was compared by one-way analysis of variance (ANOVA), with repeated measurements and post-hoc corrections. In order to determine whether the effects of HR on the PPGF were dependent on changes in BP, linear mixed model analyses for a maximum likelihood were performed. These analyses were conducted with the PPGF being regarded as the outcome variable and HR, MAP and their interactions were termed as the independent variables, with the subjects’ individual intercepts modeled as the random effects. The level of significance was set at p < 0.05. Analyses were performed using the Statistical Package for the Social Sciences (SPSS Inc., Chicago, USA) Software Version 16. Linear mixed model analyses were performed using R software [15] and the nlme package [16].

## Results

Of the 20 subjects recruited, 17 were male. Table 1 provides details of implanted pacemaker type, pacing modalities, implant indications, baseline ejection fraction and medications. Table 2 summarizes the baseline measurements of the subjects.

**Table 1:** Pacemaker and medication details.

Parameter	n
Types of Pacemaker	
Dual chamber pacemaker	17
Ventricle sensing and ventricle pacing pacemaker	3
Pacing modalities	
Atrial pacing	7
Atrioventricular pacing	7
Ventricular pacing	4
Unknown (ECG not having recorded)	2
Implant indications	
Bradycardia	8
Sick sinus syndrome	4
Heart block	5
Irregular heart rate	1
Cardiomyopathy	1
Other	1
Ejection fraction	
>60%	12
Data not available	8
Types of Medication	
Angiotensin-converting-enzyme inhibitor	5
Angiotensin receptor blocker	4
Antiarrhythmic agent	7
Anticoagulant	9
Antiplatelet therapy	3
Aspirin	4
β-blocker	7
Calcium channel blocker	5
Cardiac glycoside	2
Diuretic	2
Insulin	1
Statin	12

**Table 2:** Subjects baseline characteristics.

Parameter	Mean ± SD	Range
Age (years)	78±8	56–88
Body mass index (kg/m^2^)	26±4	17–33
Brachial systolic blood pressure (mmHg)	126±17	101–155
Brachial diastolic blood pressure (mmHg)	71±9	55–90
Brachial mean arterial pressure (mmHg)	87±10	71–106
Paced heart rate (bpm)	61±3	50–78
Total peripheral resistance (dyn.s.cm^-5^)	1293±308	644–1743
Stroke volume (ml)	85.7±25.4	38.6–136.2
Cardiac output (L/min)	5.3±1.4	3.4–7.6
PPGF (%)	50±7	39–63

PPGF, PPG fitness index.

Table 3 provides the average brachial BP, SV, CO, TPR, PPGF and the estimated vascular age at an HR of 60, 70, 80, 90 and 100 bpm. As HR increased, brachial BP, CO and estimated vascular age were significantly increased, whilst SV and PPGF were significantly decreased. Thus, a 40 bpm difference in HR of an individual from 60 to 100 bpm would result in a PPGF derived vascular age changing from 55 to 70 years old. No significant changes were observed for the TPR.

**Table 3:** Changes in cardiovascular parameters due to pacing.

Parameter	HR60	HR70	HR80	HR90	HR100	p
bSBP (mmHg)	126.75±16.96	131.05±20.41	131.15±18.23	132.15±19.86	134.90±19.40	<0.001
bDBP (mmHg)	71.65±9.11	75.70±10.71	78.55±10.35	81.70±10.40	85.95± 11.32	<0.001
bMAP (mmHg)	87.95±10.29	93.60±13.22	96.35±11.75	100.70±13.41	104.75±14.03	<0.001
SV (ml)	84.87±26.20	80.34±21.84	71.28±18.40	65.30±15.26	59.38±13.68	<0.001
CO (L/min)	5.41±1.35	5.79±1.46	5.82±1.44	5.99±1.28	6.08±1.28	0.001
TPR (dyn.s.cm^-5^)	1570.00±638.40	1506.37±585.03	1478.82±494.96	1449.89±445.65	1433.59±430.27	0.16
PPGF (%)	49.38±8.19	43.20±7.77	40.42±7.69	36.80±6.68	36.51±7.25	<0.001
Estimated vascular age (years)	55.45±8.00	62.05± 9.25	65.40±10.07	69.80±9.47	70.15±9.34	<0.001

Prefix b, brachial; SBP, systolic blood pressure; DBP, diastolic blood pressure; MAP, mean arterial pressure; SV, stroke volume; CO, cardiac output; TPR, total peripheral resistance; PPGF, PPG fitness index.

**Fig 1:**
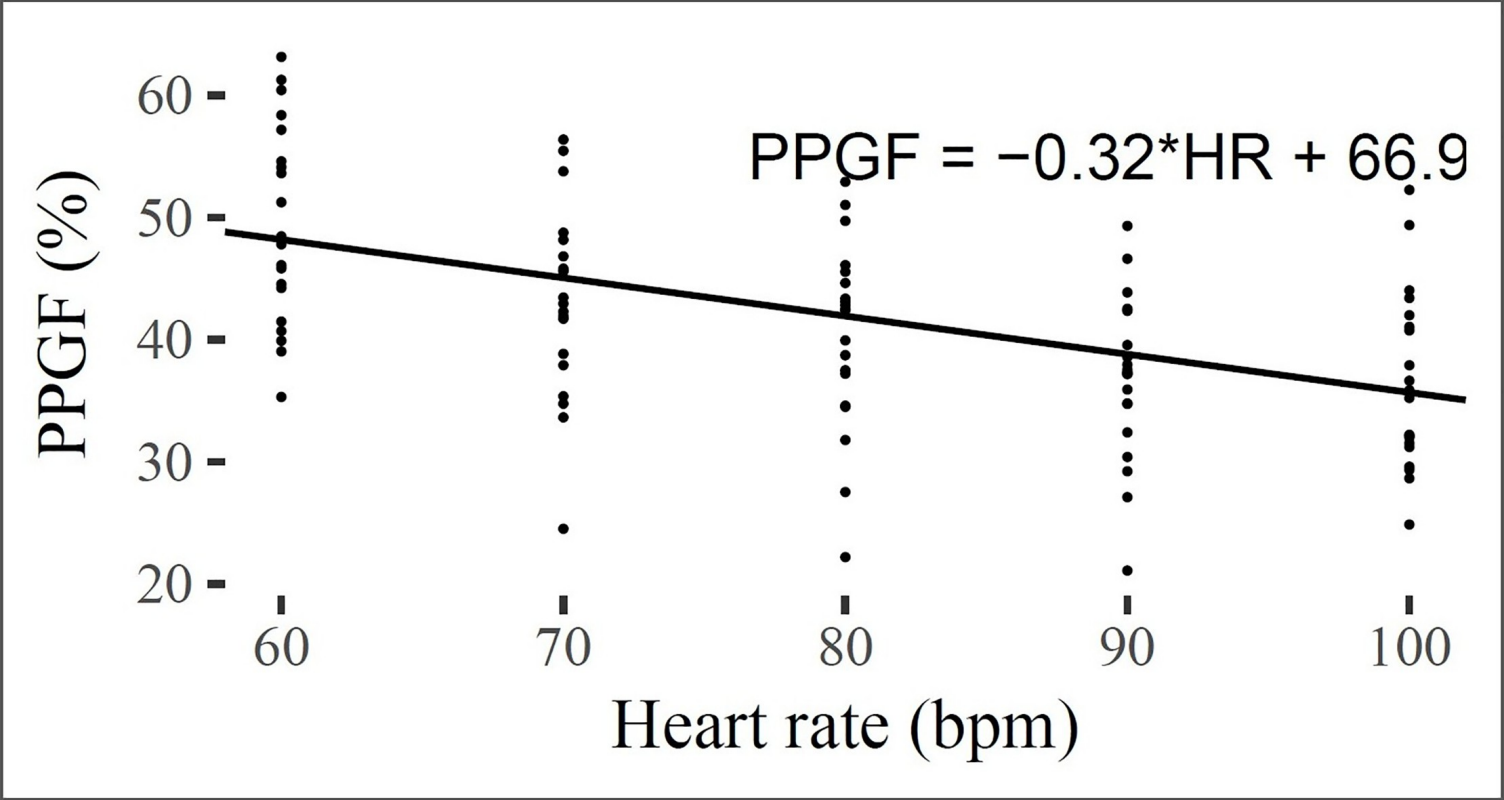
Changes in the PPGF as the HR increased.

**Fig 2:**
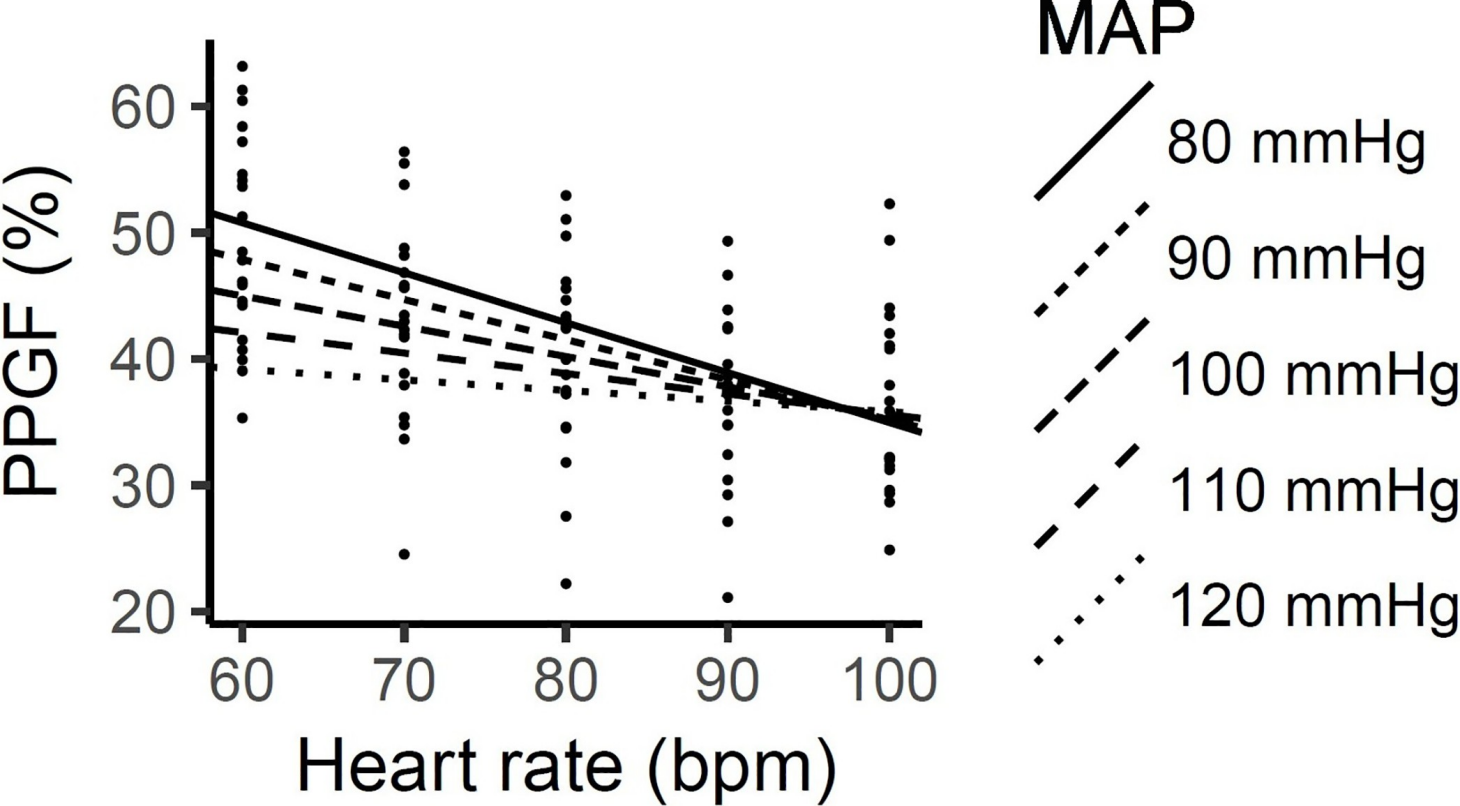
Changes in the PPGF as the HR increased at the various levels of MAP.

Table 4 shows the model parameters for effects of HR, MAP and their interactions on the PPGF, as obtained by the linear mixed model analyzes. Without the inclusion of MAP, HR had a significant negative effect on the PPGF, whereby the PPGF decreased 3.2% with each 10 bpm increase in the HR (p<0.05) (Fig 1). However, results from the mixed model analyses showed that the effect of HR on the PPGF was dependent on the BP level (Fig 2). As BP increased, the effects of HR on the PPGF were reduced.

**Table 4:** Model parameters describing the effects of HR, MAP and their interactions on the PPGF.

Model Parameters	Coefficients (± SEM)	*P*
Intercept, % (β0)	135.03 ± 23.25	< 0.001
HR, bpm (β1)	-1.02 ± 0.27	< 0.001
MAP, mmHg (β2)	-0.76 ± 0.25	0.004
HR*MAP, bpm.mmHg (β3)	0.01 ± 0.00	0.007

At any MAP level, the effect of HR can be calculated by (β3*MAP+β1)

## Discussion

### Effects of an increased HR on the Peripheral Blood Pressure, SV, CO and the TPR

This study has shown that increasing HR by cardiac pacing resulted in increase in brachial SBP and DBP. These results were in accordance with a previous study [9] and they may be related to an increase in the CO. Previous studies have found that pacing did not influence peripheral BP [17] or SBP [18]. The increase in CO may be due to an increase in HR, while SV was reduced due to shortening of ventricular filling time. An increased CO has also been observed in previous studies [19, 20, 21]. In contrast, other studies have found that increased HR did not change CO [18, 22]. The differences in subject characteristics, especially their age (young vs. old) and the presence of heart disease (cardiomyopathy, hypertensive heart disease), the types of pacemaker used (single vs. dual chamber pacemaker), as well as the method of measurements, may have affected the results. It has been shown that those with a single chamber pacemaker or with heart disease, usually had a decreased CO with pacing, when compared to those with dual-chamber pacing [23, 24] and those with a normal heart [25].

Studies on the effects of increased HR on TPR are limited in the literature. Previous studies have observed that pacing reduced TPR [20, 26], while several studies observed no change [18, 27]. Generally, TPR will decrease if pacing has adequately increased the MAP/DBP ratio or CO, as this stimulates the activation of the arterial baroreceptors with subsequent deactivation of sympathetic activity [28]. The rate of pacing might also have an effect. Sympathoexcitation has previously been found to occur at a pacing rate above 175 bpm in those with a normal left ventricular function (LVF) and at a pacing rate above 120 bpm in those with an impaired LVF. This may be due to a reduction in BP as cardiac filling time is reduced [28, 29], although HRs of that magnitude were not used in this study.

### Effects of increased HR on the PPGF

The PPGF was determined from the finger PPG, which measures the blood flow changes in the microcirculation system of the finger. Physiologically, blood flow is determined by the pressure gradient and the resistance of the blood vessels [30]. The pressure is generated by ventricular ejection and it is influenced by blood volume and arterial compliance, hence any factor that affects pressure, resistance and compliance along the arterial system may generally affect finger blood flow, and thus, the PPGF.

In this study, it was observed that the effects of HR changes on the PPGF were modulated by BP. HR was observed to have a negative effect on the PPGF at lower pressures, which gradually reduced as the BP increased. The mechanism for this observation is not well understood. However, since in this study TPR did not change with HR, the reduction in the PPGF due to HR may be mediated by a reduction in aortic compliance, in association with changes in BP. Previous studies have found that pacing increased arterial stiffness [31, 11, 7]. An increase in HR would result in a shorter duration of recoil time, resulting in vessel stiffening [32, 33]. An increased HR may also cause endothelial dysfunction [34], which may lead to a contraction of the smooth muscles of the arterial wall, and thus, resulting in an increased stiffness. A recent study has found that aortic stiffness, as measured by carotid-femoral pulse wave velocity (PWV), was an independent determinant of the PPGF and that it was negatively correlated with the PPGF [35]. This has suggested that any change in aortic stiffness may affect the PPGF. A recent study by Tan et al. observed that aortic stiffness increased as HR increased and that this effect decreased as BP increased [7]. Hence, there is a possibility that aortic stiffness also plays a role in the relationship between HR, the PPGF and BP. Further studies should be conducted in order to address this issue.

This study has also observed that vascular age varied with variations in HR. This may be due to PPGF variation, since vascular age was derived from the PPGF. As a result, any variation in the PPGF due to HR changes in individuals may affect the computed vascular age. This requires consideration when comparing PPGR-related vascular age calculations in the presence of changes in HR.

The PPGF represents one route for assessment of vascular health and it is proposed to be used as a screening method among populations. Since a change in HR can influence the PPGF, it is suggested that HR should be taken into account as a confounder of the PPGF, especially when PPGF data is to be compared within and between individuals. This could reduce the bias of comparing the PPGF in individuals with an HR variance.

### Study limitations

Several issues require consideration regarding limitations of this study: (i) This study was conducted in participants with implanted pacemakers or cardioverter defibrillators, to allow the heart to be electrically paced. As such, the study’s population was generally old, with subjects displaying cardiac complications and relying on various medications, hence the study’s findings cannot be directly extended to younger, healthier individuals. (ii) Despite the best of efforts to reproduce the physiological range of a resting heart rate (i.e., 60-100/min), the pacing induced rates that cannot be assumed to mirror results which might be obtained from variations in sinus rhythm. This limitation also applies to the observed BP correlation with the PPGF values. However, resting BP and HRs have been obtained within a physiological range. (iii) We did not take into account the effects of different types of pacing modalities to investigate effects of HR changes on PPGF. Previous studies found that atrial pacing (Ap), ventricular pacing (Vp) and sequential atrioventricular pacing (ApVp) have different impact on haemodynamic changes such as ejection duration and central pulse pressure, while no change for PWV and augmentation index [36]. Since the PPGF was also independently determined by PWV, it is suggested that changes in PPGF due to HR changes may not be so different between different types of pacing modalities. However, future studies should be conducted to address this issue. Another factor that may influenced hemodynamic changes is atrioventricular (AV) delays which was not accounted in this study. AV delays have been shown to affect BP [37, 38]. In this study, AV delays were not fixed and dependent in the individual device settings. (iv) The estimated vascular age was derived from the Malaysian population. This model will need further validation in other populations. Nevertheless, the main concern of this study was to determine the effects of HR changes on the PPGF and on vascular age, not necessarily to be used to assess the vascular risk of the subjects. (v) The values of SV, CO and TPR were derived from the finger blood pressure waveform and the look-up data built into the Finapres system. As these were not measured, there may be some bias that was introduced by the look-up system, in addition to some variability that was introduced by deriving the parameters from the finger blood pressure waveform.

## Conclusions

HR was a significant confounder of the PPGF and this factor must be taken into account in data analyses if the PPGF is to be compared between individuals with different levels of HR. The effects of HR on PPGF is also modulated by changes in BP, as well as associated changes in arterial stiffness. Future studies should be conducted in order to develop a HR-corrected PPGF and to determine the associations between the HR, PWV and the PPGF.
